# Tissue-Based Genomic Testing in Prostate Cancer: 10-Year Analysis of National Trends on the Use of Prolaris, Decipher, ProMark, and Oncotype DX

**DOI:** 10.3390/clinpract14020039

**Published:** 2024-03-19

**Authors:** Eugenio Bologna, Francesco Ditonno, Leslie Claire Licari, Antonio Franco, Celeste Manfredi, Spencer Mossack, Savio Domenico Pandolfo, Cosimo De Nunzio, Giuseppe Simone, Costantino Leonardo, Giorgio Franco

**Affiliations:** 1 Department of Urology, Rush University, Chicago, IL 60612, USA; eugenio.bologna@uniroma1.it (E.B.); francesco.ditonno@icloud.com (F.D.); leslieclaire.licari@uniroma1.it (L.C.L.); antonio.franco@uniroma1.it (A.F.); manfredi.celeste@gmail.com (C.M.); spencer_m_mossack@rush.edu (S.M.); 2 Department of Maternal-Child and Urological Sciences, Sapienza University Rome, Policlinico Umberto I Hospital, 00161 Rome, Italy; 3 Department of Urology, Azienda Ospedaliera Universitaria Integrata Verona, University of Verona, 37134 Verona, Italy; 4 Department of Urology, Sant’Andrea Hospital, Sapienza University, 00189 Rome, Italy; cosimo.denunzio@uniroma1.it; 5 Unit of Urology, Department of Woman, Child and General and Specialized Surgery, University of Campania “Luigi Vanvitelli”, 80138 Naples, Italy; 6 Department of Neurosciences, Reproductive Sciences and Odontostomatology, University of Naples “Federico II”, 80138 Naples, Italy; pandolfosavio@gmail.com; 7 Department of Urology, “Regina Elena” National Cancer Institute, 00144 Rome, Italy; puldet@gmail.com (G.S.); costantino.leonardo@gmail.com (C.L.)

**Keywords:** PCa, genetic tests, active surveillance, adjuvant therapies, AS, aRT

## Abstract

Background: Prostate cancer (PCa) management is moving towards patient-tailored strategies. Advances in molecular and genetic profiling of tumor tissues, integrated with clinical risk assessments, provide deeper insights into disease aggressiveness. This study aims to offer a comprehensive overview of the pivotal genomic tests supporting PCa treatment decisions, analyzing—through real-world data—trends in their use and the growth of supporting literature evidence. Methods: A retrospective analysis was conducted using the extensive PearlDiver™ Mariner database, which contains de-identified patient records, in compliance with the Health Insurance Portability and Accountability Act (HIPAA). The International Classification of Diseases (ICD) and Current Procedural Terminology (CPT) codes were employed to identify patients diagnosed with PCa during the study period—2011 to 2021. We determined the utilization of primary tissue-based genetic tests (Oncocyte DX^®^, Prolaris^®^, Decipher^®^, and ProMark^®^) across all patients diagnosed with PCa. Subsequently, within the overall PCa cohort, patients who underwent radical prostatectomy (RP) and received genetic testing postoperatively were identified. The yearly distribution of these tests and the corresponding trends were illustrated with graphs. Results: During the study period, 1,561,203 patients with a PCa diagnosis were recorded. Of these, 20,748 underwent tissue-based genetic testing following diagnosis, representing 1.3% of the total cohort. An increasing trend was observed in the use of all genetic tests. Linear regression analysis showed a statistically significant increase over time in the use of individual tests (all *p*-values < 0.05). Among the patients who underwent RP, 3076 received genetic analysis following surgery, representing 1.27% of this group. Conclusions: Our analysis indicates a growing trend in the utilization of tissue-based genomic testing for PCa. Nevertheless, they are utilized in less than 2% of PCa patients, whether at initial diagnosis or after surgical treatment. Although it is anticipated that their use may increase as more scientific evidence becomes available, their role requires further elucidation.

## 1. Introduction

Management of prostate cancer (PCa) has significantly evolved over the years; assessing the trade-offs between the benefits and harms of treatments for localized PCa is increasingly recognized as crucial in therapeutic decision-making [1,2]. 

The focus in PCa has shifted to the “when and if” of treatment rather than the “how.” This shift is particularly relevant in two critical phases: deciding between active treatment or active surveillance (AS) following the histological diagnosis of PCa, and considering adjuvant therapy after radical prostatectomy (RP). 

In recent years, the eligibility criteria for AS have undergone significant changes; factors such as disease stage, prostate-specific antigen (PSA) values, core involvement at biopsies, Gleason Score (GS), and comprehensive risk score classification [3,4] have been re-evaluated to expand the cohort of patients suitable for active monitoring.

Despite broader eligibility, over 40% of low-risk patients in the United States undergo immediate treatment, leading to a significant number undergoing unnecessary treatment [5].

An opposite situation can be observed after RP. Various studies have shown the benefits of adjuvant therapy (radiotherapy with or without androgen deprivation therapy) in specific patient subgroups, supported by improved progression-free [6] and metastasis-free survival rates [7]. However, the side effects of adjuvant treatments, especially during the critical early post-operative period, limit the broader application of these treatments [8,9]. 

In this evolving landscape, the approach to PCa therapy is moving towards patient-tailored strategies. Molecular and genetic analyses of tumor tissues, combined with clinical risk factors, aim to provide deeper insights into disease aggressiveness [10,11,12].

Several tissue-based prognostic markers have been developed for clinical use and are now commercially available. 

The Oncotype^®^ DX Genomic Prostate Score (GPS) is a quantitative real-time polymerase chain reaction (PCR) assay applied to fixed paraffin-embedded tissue samples from needle prostate biopsies (PB). It evaluates a 17-gene signature across four biological pathways: androgen signaling, cellular organization, stromal response, and cellular proliferation, in addition to five housekeeping genes. GPS scores range from 0 to 100, with higher scores indicating increased cancer aggressiveness [13].

The Genomic Classifier (GC) Decipher^®^ test was developed and validated through a retrospective analysis of 639 RP patients from the Mayo Clinic registry [14]. Utilizing a microarray system to analyze 1.4 million genomic markers, it defines a 22-gene signature involved in several cellular processes like cell proliferation, differentiation, immune response modulation, and modulation of androgen-signaling pathways. The Decipher test provides a quantifiable risk assessment, scoring from 0 to 1, which correlates with the likelihood of subsequent adverse clinical outcomes, including biochemical recurrence (BCR) and early clinical metastases post-RP.

The Prolaris^®^ test is a tissue-based RT-PCR assay measuring the expression of 31 cell-cycle progression (CCP) genes and 15 housekeeping genes. The output is a proliferative index—ranging from 0 to 10—expressed as a CCP score, which correlates with various clinical outcomes [15].

Lastly, the ProMark^®^ test is an advanced, automated multiplex immunofluorescence in situ imaging method that quantitatively evaluates eight protein biomarkers and their activation states in PB tissues [16]. Initially characterized by a panel of 12 signature protein biomarkers aimed at predicting PCa aggressiveness and lethality [17], the ProMark test was later refined to an 8-protein marker panel. This updated panel showed an improved predictive ability to differentiate between favorable and unfavorable pathology at RP compared to clinical risk stratification alone [16].

These prognostic tests seek to improve tumor characterization and narrow the gap between clinical guidelines and actual clinical practice. 

Our study aims to provide an overview of the primary genomic tests influencing PCa management and evaluate their utilization trends based on real-world data.

## 2. Materials and Methods

### 2.1. Dataset

The dataset for this analysis was sourced from the extensive PearlDiver^®^ Mariner database (PearlDiver Technologies, Colorado Springs, CO, USA), a HIPAA-compliant, anonymized national repository of insurance billing records [18]. This resource catalogs healthcare interactions across both inpatient and outpatient settings, enabling the longitudinal study of patient trajectories. The database encompasses claims data for a diverse cohort of over 150 million distinct individuals, compiled from 2011 to 2021, and includes detailed reimbursement information spanning facilities, practitioners, ancillary services, and pharmacies. Its coverage is comprehensive, extending to all payer models across the entirety of the U.S. states and territories. 

Utilization of the database is enabled through the application of International Classification of Diseases (ICD) codes, both 9th and 10th editions, along with Current Procedural Terminology (CPT) codes. Data integrity is ensured via rigorous audits and review processes by independent third parties [18]. It is noteworthy that a significant portion of the commercial insurance claims recorded in the database predominantly originate from Humana and United Healthcare [19].

### 2.2. Study Population Statistical Analyses

As a secondary analysis of deidentified data, the study was granted exempt status by the Institutional Review Board (IRB). We successfully identified subjects diagnosed with PCa from 2011 to 2021 using relevant ICD-9 and ICD-10 diagnosis codes.

Demographic variables included age, region, obesity, diabetes mellitus, and Charlson Comorbidity Index (CCI). Additionally, we evaluated the prevalence of Social Determinants of Health (SDOH), such as access to education, quality of healthcare, conditions of the neighborhood and built environment, social and community context, and economic stability. 

Subsequently, we determined the utilization of primary tissue-based genetic tests (Oncotype DX^®^—Genomic Health, Redwood City, CA, USA; Prolaris^®^—Myriad Genetics, Salt Lake City, UT, USA; Decipher^®^—Decipher Biosciences, San Diego, CA, USA; ProMark^®^—Metamark Genetics Inc., Durham, NC, USA) in patients diagnosed with PCa.

Within the overall PCa population, we specifically identified those who underwent RP. The methodology for identifying patients within this subgroup who received genetic testing post-surgery was consistent with the approach taken for the overall cohort. 

We also evaluated the annual distribution of these tests within both patient groups throughout the study period, computing percentages and illustrating the trends with corresponding graphs. Owing to the relatively small number of patients who underwent RP and were subsequently tested with Oncotype DX^®^ and Prolaris^®^, we limited our graphical presentations to trends for Decipher^®^ and ProMark^®^ tests only.

Linear regression analysis was then employed to statistically quantify the trend patterns for each test during the study period among the overall PCa patients.

Finally, multivariable logistic regression was utilized to assess factors associated with the likelihood of receiving a genetic test.

## 3. Results

Throughout the study period, a total of 1,561,203 patients with PCa diagnoses were recorded. Of this cohort, 241,445 patients underwent RP. The baseline characteristics of both groups are summarized in Table 1. 

**Figure 1:**
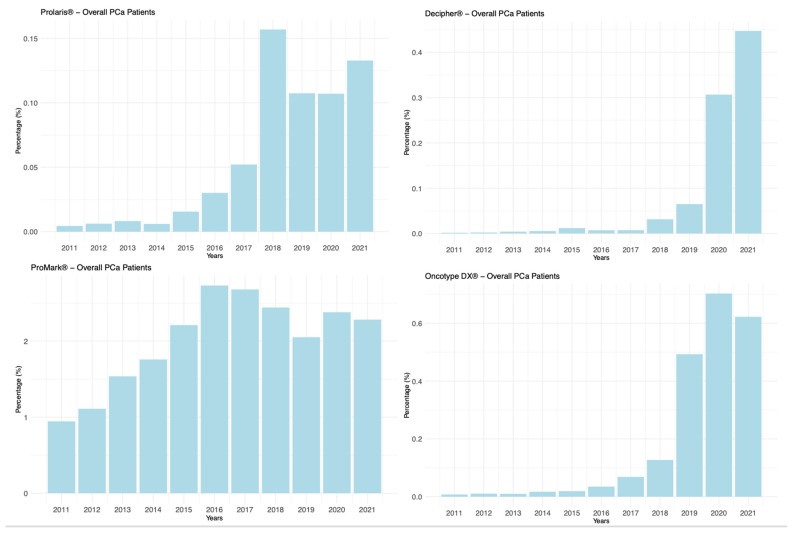
Bar Plot Depicting Utilization Trends of Four Tissue-Based Genomic Tests Over Study Period in Overall PCa Patients.

Among the patients diagnosed with PCa, 20,748 were subjected to tissue-based genetic testing following diagnosis, constituting 1.3% of the overall cohort. Figure 1 illustrates the annual percentage trend in the prescription of the genetic tests under study—Oncotype DX^®^, Prolaris^®^, Decipher^®^, and ProMark^®^—over the period considered. A rising trend was observed for all genetic tests. 

Linear regression analysis revealed a statistically significant increase in the use of each individual test (all *p*-values < 0.05) (Table 2). All tests showed a positive regression coefficient, indicating increased usage over time. Notably, ProMark^®^ displayed the most significant annual increase, with the highest regression coefficient (0.1375), while Prolaris^®^ exhibited the best model fit (adjusted R^2^ = 0.7397).

**Figure 2:**
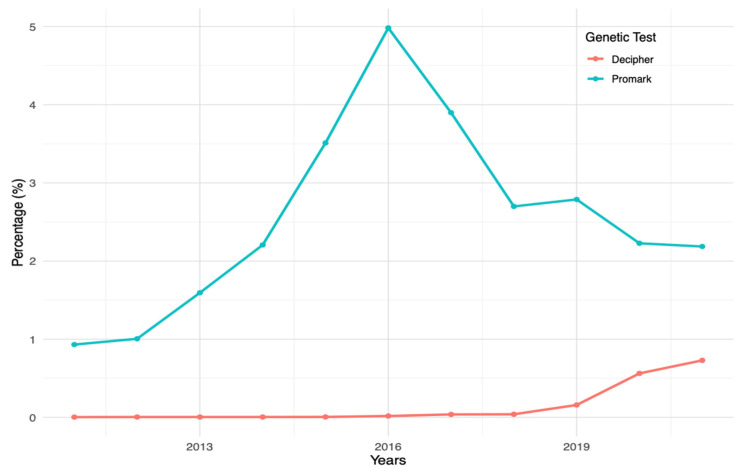
Trends in the Use of Decipher^®^ and Promark^®^ after Radical Prostatectomy Over the Study Period (2011–2021).

Among the patients who underwent RP, 3076 received genetic analysis following surgery, representing 1.27% of the surgical subset. Figure 2 displays the percentage of usage for Decipher^®^ and ProMark^®^ within this subgroup throughout the study period.

In the adjusted multivariate regression analysis, age was inversely associated with the probability of undergoing genetic testing, with each additional year decreasing the odds by 3.5% (adjusted odds ratio [OR] = 0.965, 95% confidence interval [CI]: 0.963–0.966, *p* < 0.001). SDOH and CCI were not associated with significant changes in the likelihood of receiving genetic testing (SDOH adjusted OR = 0.90, 95% CI: 0.794–1.023, *p* = 0.118; CCI adjusted OR = 1.001, 95% CI: 0.996–1.007, *p* = 0.475).

## 4. Discussion

Our analysis provides interesting insights into the utilization and impact of tissue-based genomic testing in PCa management. We found that over the 10-year study period, only 1.32% of patients underwent genomic testing after a PCa diagnosis. 

However, the implementation of genomic tumor profiling has shown a steady increase, mirroring the growth in scientific evidence that supports its clinical use. 

This burgeoning evidence has also influenced guidelines by the National Comprehensive Cancer Network (NCCN) [20] and the European Association of Urology (EAU) [4], which now acknowledge the value of tests such as Decipher^®^, Prolaris^®^, Oncotype^®^, and ProMark^®^ in PCa management. Specifically, the use of these additional tests is recommended for patients in whom “the assay result, when considered as a whole with routine clinical factors, is likely to affect management.” 

Table 3 summarizes the key characteristics of the tests under study, highlighting their specific indications and relevant clinical implications.

Our study shows a low adoption prior to 2015, followed by a progressive increase in the use of all tests, with Oncotype^®^ DX (GPS) and Decipher^®^ (GC) exhibiting a notable yet gradual rise. 

Genomic tests are instrumental in refining patient selection for AS, with their full clinical benefits expected to unfold over time. Consequently, the observed increase in their use could reflect a more efficient decision-making process by physicians and patients, aligning with long-term health outcomes rather than immediate clinical and prognostic improvements. 

Several factors could account for this overall ascending trend. Foremost, the growing body of published literature endorsing the efficacy of these tests may have bolstered their acceptance within the medical community. Additionally, enhanced accessibility in clinical settings could have facilitated increased usage. Finally, the marketing strategies employed by the test providers could influence the adoption rate among healthcare professionals, and the price variability among different tests could also be a significant factor.

Our study has also demonstrated an association between older age and a decreased likelihood of being recommended for genetic testing. These findings suggest that urologists are more likely to utilize the additional prognostic information provided by genetic tests in the decision-making process for younger patients [21]. This is attributed to younger patients’ longer life expectancy, which allows for a more informed decision-making process that carefully weighs the potential harms and benefits of the different therapeutic options. 

Specifically focusing on the preference for the ProMark^®^ test observed in our study, we posit that this test’s ease of integration into existing diagnostic workflows could be a key factor—its results do not depend on other clinical or diagnostic data, making it a straightforward option for clinicians. However, the ProMark^®^ test is linked to a less specific CPT code, which might result in a broader categorization of various genetic tests under this code. This necessitates a cautious interpretation of this finding, as the data may encompass a wider range of tests than intended.

Peabody et al. conducted a randomized clinical utility trial to assess the impact of the ProMark^®^ test on the decision for active treatment versus AS in simulated cases of ISUP 1 and ISUP 2 PCa [22]. The additional clinical information derived from the test results led to nearly a 30% reduction in recommendations for active treatment in low- and favorable intermediate-risk PCa.

Further analyzing the observed trends, the uptick in the utilization of Oncotype DX^®^ (GPS) and Decipher^®^ tests may be linked to their applicability to a wider patient demographic (Table 3).

Originally, the GPS was conceived and validated for its potential utility in managing tumor heterogeneity and biopsy undersampling [13]. Klein et al. demonstrated that a 20-point increase in the GPS score was predictive of both high-grade disease (odds ratio [OR] = 2.3, *p* < 0.001) and high-stage disease (OR = 1.9, *p* = 0.003) at surgical pathology. 

Similar results were reported by Kornberg et al., who evaluated the suitability for AS of different patient subgroups: International Society of Urological Pathology (ISUP) grade 1, low-volume ISUP grade 2 (33% or fewer positive cores), clinical stage T1/T2, PSA < 20 ng/mL, and a Cancer of the Prostate Risk Assessment (CAPRA) score < 6 [23]. A 5-unit increase in the GPS score was significantly associated with an elevated risk of adverse pathology (AP) (hazard ratio [HR] 1.16, *p* < 0.01) and an increased risk of biochemical recurrence (BCR) (HR 1.10, *p* = 0.04).

However, a multicenter study with a cohort of 432 patients, of whom 101 underwent RP, did not find a correlation between the GPS and AP in multivariate analysis, nor was there an association with subsequent biopsy upgrades (*p* = 0.48) [24]. 

Conversely, a 2019 multicenter prospective study validated the GPS score’s predictive role for AP (OR 2.2 and 1.9 for every 20-unit increase in univariate and multivariate analyses, respectively) and reported an association with reduced decisional conflict among patients concerning treatment decisions [25].

In 2021, Murphy et al. reported the outcomes of a randomized trial assessing the impact of the GPS in 200 patients with low and low-intermediate risk PCa who were randomly assigned to receive standard counseling with or without the GPS assay [26]. The authors highlighted two interesting findings: despite a high baseline acceptance of AS, the GPS assay did not further increase its acceptance, nor did it seem to enhance the perceived quality of the patients’ decision-making. 

In this context, Eymach et al. addressed the psychological distress associated with AS, describing a condition that profoundly affects patients. This distress is characterized by an awareness of their disease—even if classified as low-risk—and is intensified by the anxiety of frequent check-ups [27]. Thus, the decision between AS and active treatment remains complex for patients, despite the additional prognostic information provided by genomic tests. It could be speculated that the hoped-for increase in the indication for AS may be more a result of increased “confidence” among physicians in proposing active monitoring than due to easier acceptance by the patient. 

Previous reports, however, have provided additional insights. In the prospective study by Eure et al.—among patients with clinically low-risk PCa—GPS led to a 23% refinement of individual risk, a 22% increase in the selection of AS after diagnosis, and a 21% increase in the retention of patients on AS one year from the initial decision [28]. Additionally, the authors described greater confidence reported by the physician and reduced decisional conflict reported by patients after GPS testing. 

Similar findings were presented by Badani et al. [29]. The authors reported a 24% increase in AS recommendations and increased physician confidence due to the risk refinement provided by GPS. It therefore seems appropriate to consider an important role for genomic tests in boosting physicians’ confidence in proposing an active monitoring regime, which is reflected in improved decision-making quality among patients; this could also significantly impact the psychological well-being of patients on the active surveillance pathway. 

The Decipher^®^ test (GC) has also been evaluated as a supportive tool for improved decision-making in AS. Herlemann et al. assessed the GC’s predictive capacity for AP at the time of RP in men with NCCN favorable-intermediate risk PCa [30]. They reported a heightened risk of AP (OR = 6.8, *p* < 0.001) in patients with high-risk Decipher scores (GC > 0.60), suggesting its utility in refining patient selection for AS.

As for the Prolaris^®^ test, Tosoian et al.’s 2017 study provided evidence that the CCP score is beneficial for stratifying clinical risk among patients classified as NCCN low-risk [31]. The study highlighted the CCP score’s capacity to enhance the stratification process for identifying patients with a higher risk of BCR. This refinement could potentially improve the selection of candidates for AS and inform more comprehensive treatment decision-making processes.

The in-depth insights provided by genomic tests are also fundamental in selecting adjuvant therapies after RP. NCCN guidelines recommend adjuvant radiotherapy (RT)—with or without androgen deprivation therapy (ADT)—in cases where adverse pathological or laboratory characteristics are found after radical treatment [20,32]. 

In the RADICALS-RT trial, patients with at least one risk factor for BCR were randomly assigned to receive either adjuvant RT or undergo an observation policy with salvage RT for PSA biochemical progression [33]. The results indicated that the 5-year biochemical progression-free survival was not significantly different between the two groups. However, a worsening of self-reported urinary incontinence was noted in the adjuvant RT group. These findings justify the limited use of adjuvant RT in clinical practice [34], as the risk of overtreatment may result in RT-induced toxicity and potentially compromise the functional outcomes associated with surgery [35].

In this patient setting, characterized by contrasting evidence in the literature and a high risk of overtreatment, the NCCN guidelines suggest that “molecular assay should be considered if not previously performed to inform adjuvant treatment if adverse features are found post-radical prostatectomy.” 

In our study, only 1.27% of patients underwent genomic testing following RP. Although the utilization rate in this setting is low, it is important to note that the percentages of genomic test adoption post-diagnosis and post-RP are similar. This observation is noteworthy, considering that there is more evidence supporting the use of genomic testing to guide eligibility for AS than for post-surgical decisions.

This may suggest that determining the indication for adjuvant RT treatment poses a great clinical challenge for physicians, requiring a careful evaluation of the risks and benefits. Genomic testing, therefore, could provide crucial support in making decisions that reflect the disease’s intrinsic aggressiveness rather than relying solely on clinical and laboratory data. 

Specifically, the NCCN guidelines recommend the Decipher^®^ GC test after surgery, which may support the rising trend observed in the present study, along with a concurrent reduction in the use of the ProMark test^®^ (Figure 2). 

Additionally, recent trial results have highlighted the relevance of genomic testing in this patient setting. A randomized phase III trial assessed patients who had undergone RP and were receiving dose-escalated salvage RT using the Decipher^®^ GC test [36]. This test, differentiating between high and low-intermediate risk, was independently associated with biochemical progression (HR 2.26, *p*-value = 0.003), clinical progression (HR = 2.29, *p*-value = 0.003), and an increased use of ADT (HR = 2.99, *p*-value 0.001).

A recent meta-analysis led by Spratt et al. encompassed a collective sample of 855 patients from five different studies, elucidating GC’s role in predicting the cumulative 10-year incidence of metastasis [37]. Patients were stratified into low, intermediate, and high-risk categories based on GC score thresholds of 0.45 and 0.60. The analysis revealed respective 10-year cumulative metastasis incidences of 5.5%, 15.0%, and 26.7% across each risk group. 

Interestingly, Dalela et al. conducted a study to determine the efficacy of the Decipher^®^ test in selecting patients for adjuvant treatment after RP [38]. Utilizing a four-item risk stratification tool—which includes pT3b/T4 disease, pathological Gleason score 8–10, lymph node invasion, and a GC score > 0.6—they demonstrated a significant reduction in the 10-year clinical recurrence rate with adjuvant RT for patients with a GC risk score of 2 or greater. This underscores the role of the Decipher^®^ test, in conjunction with adverse clinical factors, in optimizing post-RP adjuvant treatment decisions. 

Clinical trials are currently underway to assess the Decipher^®^ test’s role in tailoring adjuvant ADT plus RT protocols following RP in patients with high-risk [39] and unfavorable intermediate-risk PCa [40].

As for the Prolaris^®^ test, Cuzick et al. initially evaluated the CCP score in patients who underwent RP and transurethral resection of the prostate (TURP) [15]. The CCP score emerged as the strongest multivariate predictor of BCR following RP, with the risk nearly doubling for each unit increase in the score (HR = 1.7; *p* < 0.001). 

Subsequent studies have affirmed the clinical validity of the Prolaris^®^ test in prostate biopsy samples. Cuzick et al. demonstrated a two-fold increase in the risk of PCa-specific mortality per unit increase in the CCP score (HR = 2.02; *p* < 0.001) [41]. Bishoff et al. corroborated the prediction of BCR (HR = 1.47; *p* < 0.001) and, although based on a limited number of events, reported the CCP score as the strongest predictor of metastatic disease (HR = 4.19; *p* < 0.001) [42]. 

Finally, Saad et al. assessed the correlation of the ProMark^®^ score with the risk of BCR in 288 men prior to RP [43]. The assay scores were predictive of BCR on univariate analysis (HR 1.724, *p* = 0.0002 per 20% score change), outperforming other preoperative parameters. Moreover, combining the assay score with the NCCN clinical stage yielded a higher prognostic value (HR 1.579, *p* = 0.0017 per 20% score change) than staging alone.

Our study is not without limitations. Firstly, although we carefully selected codes for data extraction, the use of CPT codes to identify genomic tests in clinical practice may not have been entirely accurate. Consequently, the data presented should be viewed globally rather than focusing on specific tests. Additionally, incorrect CPT coding in clinical practice might have led to the exclusion of patients who actually underwent these tests, potentially causing an underestimation of the real numbers. It must also be emphasized, as part of the limitations, that the ProMark^®^ test is associated with a less specific CPT code, which increases the likelihood that other genetic tests may be included in the numerical estimate for ProMark^®^. Another inherent limitation is the PearlDiver Mariner database’s lack of information on patients’ race, tumor characteristics, and treatment protocols, which hinders a more comprehensive analysis of the results. 

Despite these limitations, we believe that our findings, combined with a state-of-the-art overview of genomic testing, provide a comprehensive perspective on the integration of these tests into clinical practice over recent years.

## 5. Conclusions

Our analysis indicates a rising trend in the use of tissue-based genomic testing for PCa. Nevertheless, they are used in less than 2% of PCa patients, either at the initial diagnosis or after surgical treatment. While it is anticipated that their usage might increase as additional scientific evidence becomes available, the precise role of these tests in clinical practice remains to be further defined.

## Figures and Tables

**Table 1:** Characteristics of Prostate Cancer Patients at Diagnosis and Following Radical Prostatectomy (RP).

Variable	Prostate Biopsy Patients (n = 1,561,203)	RP Patients (n = 241,445)
Age, years, Mean ± SD	68.51 ± 8.33	64.39 ± 7.60
Region, n (%)		
Midwest	352,299 (22.5)	63,632 (26.3)
Northeast	362,326 (23.2)	49,380 (20.4)
South	620,196 (39.7)	92,368 (38.3)
West	220,271 (14.2)	35,127 (14.5)
Unknown	6111 (0.4)	938 (0.5)
Charlson Comorbidity Index, Mean ± SD	2.79 ± 2.64	2.85 ± 2.57
Obesity, n (%)	473,656 (30.3)	82,082 (33.9)
Diabetes, n (%)	411,863 (26.4)	54,763 (22.7)
Social Determinants of Health (SDOH), n (%)		
Lack of Education Access and Quality	460 (0.03)	62 (0.03)
Inadequate Health Care Access and Quality	241 (0.02)	22 (0.01)
Poor Neighborhood and Built Environment	4305 (0.28)	747 (0.31)
Negative Social and Community Context	10,209 (0.65)	1503 (0.62)
Economic instability	5070 (0.32)	604 (0.25)
Overall	19,451 (1.25)	2836 (1.17)
Use of Tissue-based genomic Testing, n (%)	20,748 (1.32)	3076 (1.27)

Abbreviations: RP (Radical Prostatectomy), SD (Standard Deviation).

**Table 2:** Linear Regression Trends of Tissue-Based Genomic Testing in Overall Prostate Cancer Patients.

Genetic Test	Regression Coefficient (Slope)	Model Fit (Adjusted R^2^)	*p*-Value
Prolaris^®^	0.0152	0.7397	0.0004
Oncotype DX^®^	0.0687	0.6677	0.0013
Decipher^®^	0.0334	0.4888	0.01
Promark^®^	0.1375	0.5248	0.007

**Table 3:** Summary of Key Characteristics of Tissue-Based Genetic Tests for PCa.

Genetic Test	Clinical Indication	Testing Method	Assessed Parameters	Scoring	Clinical Implications	Other Characteristics
Prolaris^®^	After biopsy: NCCN very low low, favorable intermediate-risk localized prostate cancer After RP: patients who may benefit from aggressive intervention/at high risk of recurrence	Reverse transcriptase PCR	Gene activity related to cell cycle: 46 genes (31 Cell Cycle Progression + 15 housekeeping genes)	Cell Cycle Progression (CCP) score between 0 and 10 Higher scores indicative of more aggressive disease	Provides risk assessment to aid treatment choice between AS, single modal or multi-modal treatment Provides: 10-y risk (%) of DSM with conservative treatment 10-y risk (%) of metastasis with RT or RP 10-y risk (%) of metastasis with RT + ADT	Result combined with patient’s clinical data (CAPRA score and NCCN)
Decipher^®^	After biopsy: all GS, all PSA values, all Stages After RP: patients with adverse pathology, all PSA values (including undetectable, rising, and persistently elevated PSA)	Microarray genomic testing	Expression of 22 coding and noncoding RNAs	Genomic Risk (GR) Score between 0 and 1 Higher scores indicative of more aggressive disease	After biopsy: High risk (>0.6): patients may benefit from treatment intensification with multimodal therapy Low risk (<0.45): patients can be candidates for AS Provides: 5-y and 10-y risk (%) of metastasis with RT or RP 15-y Risk (%) of PCa mortality with RT or RP Risk (%) of adverse pathology at RP based on biopsy After RP: High risk (>0.6): patients may benefit from RT with concurrent ADT; patients may benefit from earlier, more intense, or multimodality therapy, and may consider clinical trials of novel therapies Provides: 5-y and 10-y risk (%) of metastasis 15-y Risk (%) of DSM	Result not combined with other clinical or pathologic parameters Additional information: After biopsy: Personalized risk of metastasis if combined with patient’s NCCN risk category After RP: Personalized 10-y risk of metastasis if combined with patient’s clinical and pathologic features RT timing Personalized 5-y risk of metastasis after post-RP RT Treatment intensity: RT + ADT? Personalized 5-y risk of progression to ADT after Radiotherapy alone
ProMark^®^	NCCN very low, low and intermediate risk localized prostate cancer	Proteomic analysis	Quantify the values of 8 tumor progression-related biomarker proteins	ProMark Score between 0 and 100 Higher scores indicative of more aggressive disease	Provides risk assessment to aid treatment choice between AS and active treatment Predicts BCR in patients after RP Provides: Risk (%) of aggressive disease	Result not combined with other clinical or diagnostic data (NCCN, CAPRA, D’Amico) Additional information: Likelihood (%) of Adverse Pathology at RP Personalized risk of aggressive disease if combined with patient’s NCCN risk category
Oncotype DX^®^	NCCN low, intermediate, and high-risk localized prostate	Reverse transcriptase PCR	Expression of 17 genes (12 cancer-related and 5 reference genes)	Genomic Prostate Score (GPS) between 0 and 100 Higher scores indicative of more aggressive disease	Low risk patients: help inform the AS decision High Risk patients: help inform the treatment intensity decision Provides: Low risk patients: Likelihood (%) of Adverse Pathology at RP High Risk patients: Likelihood (lower\higher) of disease progression	Result combined to NCCN risk group Additional information: 10-y likelihood (%) of Metastasis after RP 10-y likelihood (%) of DSM after RP

Abbreviations: PCR (Polymerase Chain Reaction), DSM (Disease-Specific Mortality), RT (Radiotherapy), RP (Radical Prostatectomy), ADT (Androgen Deprivation Therapy), CAPRA (Cancer of the Prostate Risk Assessment), NCCN (National Comprehensive Cancer Network), GS (Gleason Score), PSA (Prostate-Specific Antigen), AS (Active Surveillance), PCa (Prostate Cancer).

## Data Availability

The PearlDiver database is a private national database that requires private access to a password-protected server.

## References

[1] Hamdy F.C., Donovan J.L., Lane J.A., Metcalfe C., Davis M., Turner E.L., Martin R.M., Young G.J., Walsh E.I., Bryant R.J. (2023). Fifteen-Year Outcomes after Monitoring, Surgery, or Radiotherapy for Prostate Cancer. N. Engl. J. Med..

[2] Tohi Y., Kato T., Sugimoto M. (2023). Aggressive Prostate Cancer in Patients Treated with Active Surveillance. Cancers.

[3] Schaeffer E.M., Srinivas S., Adra N., An Y., Barocas D., Bitting R., Bryce A., Chapin B., Cheng H.H., D’amico A.V. (2023). Prostate Cancer, Version 4.2023, NCCN Clinical Practice Guidelines in Oncology. J. Natl. Compr. Cancer Netw..

[4] Mottet N., van den Bergh R.C.N., Briers E., van den Broeck T., Cumberbatch M.G., De Santis M., Fanti S., Fossati N., Gandaglia G., Gillessen S. (2020). EAU-EANM-ESTRO-ESUR-SIOG Guidelines on Prostate Cancer—2020 Update. Part 1: Screening, Diagnosis, and Local Treatment with Curative Intent. Eur. Urol..

[5] Cooperberg M.R., Meeks W., Fang R., Gaylis F.D., Catalona W.J., Makarov D.V. (2023). Time Trends and Variation in the Use of Active Surveillance for Management of Low-risk Prostate Cancer in the US. JAMA Netw. Open.

[6] Bolla M., van Poppel H., Tombal B., Vekemans K., Da Pozzo L., de Reijke T.M., Verbaeys A., Bosset J.-F., van Velthoven R., Colombel M. (2012). Postoperative radiotherapy after radical prostatectomy for high-risk prostate cancer: Long-term results of a randomised controlled trial (EORTC trial 22911). Lancet.

[7] Thompson I.M., Tangen C.M., Paradelo J., Lucia M.S., Miller G., Troyer D., Messing E., Forman J., Chin J., Swanson G. (2009). Adjuvant Radiotherapy for Pathological T3N0M0 Prostate Cancer Significantly Reduces Risk of Metastases and Improves Survival: Long-Term Followup of a Randomized Clinical Trial. J. Urol..

[8] Kneebone A., Fraser-Browne C., Duchesne G.M., Fisher R., Frydenberg M., Herschtal A., Williams S.G., Brown C., Delprado W., Haworth A. (2020). Adjuvant radiotherapy versus early salvage radiotherapy following radical prostatectomy (TROG 08.03/ANZUP RAVES): A randomised, controlled, phase 3, non-inferiority trial. Lancet Oncol..

[9] Del Giudice F., Huang J., Li S., Sorensen S., Enemchukwu E., Maggi M., Salciccia S., Ferro M., Crocetto F., Pandolfo S.D. (2022). Contemporary trends in the surgical management of urinary incontinence after radical prostatectomy in the United States. Prostate Cancer Prostatic Dis..

[10] Franco A., Autorino R. (2023). ExoDx test for prostate cancer: The future is liquid—Editorial Comment. Prostate Cancer Prostatic Dis..

[11] Di Lorenzo G., Autorino R., D’Armiento F., Mignogna C., De Laurentiis M., De Sio M., D’Armiento M., Damiano R., Vecchio G., De Placido S. (2004). Expression of proto-oncogene c-kit in high risk prostate cancer. Eur. J. Surg. Oncol. (EJSO).

[12] Moreno C.S., Winham C.L., Alemozaffar M., Klein E.R., Lawal I.O., Abiodun-Ojo O.A., Patil D., Barwick B.G., Huang Y., Schuster D.M. (2023). Integrated Genomic Analysis of Primary Prostate Tumor Foci and Corresponding Lymph Node Metastases Identifies Mutations and Pathways Associated with Metastasis. Cancers.

[13] Klein E.A., Cooperberg M.R., Magi-Galluzzi C., Simko J.P., Falzarano S.M., Maddala T., Chan J.M., Li J., Cowan J.E., Tsiatis A.C. (2014). A 17-gene Assay to Predict Prostate Cancer Aggressiveness in the Context of Gleason Grade Heterogeneity, Tumor Multifocality, and Biopsy Undersampling. Eur. Urol..

[14] Erho N., Crisan A., Vergara I.A., Mitra A.P., Ghadessi M., Buerki C., Bergstralh E.J., Kollmeyer T., Fink S., Haddad Z. (2013). Discovery and Validation of a Prostate Cancer Genomic Classifier that Predicts Early Metastasis Following Radical Prostatectomy. PLoS ONE.

[15] Cuzick J., Swanson G.P., Fisher G., Brothman A.R., Berney D.M., Reid J.E., Mesher D., Speights V.O., Stankiewicz E., Foster C.S. (2011). Prognostic value of an RNA expression signature derived from cell cycle proliferation genes in patients with prostate cancer: A retrospective study. Lancet Oncol..

[16] Blume-Jensen P., Berman D.M., Rimm D.L., Shipitsin M., Putzi M., Nifong T.P., Small C., Choudhury S., Capela T., Coupal L. (2015). Development and Clinical Validation of an In Situ Biopsy-Based Multimarker Assay for Risk Stratification in Prostate Cancer. Clin. Cancer Res..

[17] Shipitsin M., E Small C., Choudhury S., Giladi E., Friedlander S.F., Nardone J., Hussain S., Hurley A.D., Ernst C., Huang Y.E. (2014). Identification of proteomic biomarkers predicting prostate cancer aggressiveness and lethality despite biopsy-sampling error. Br. J. Cancer.

[18] Research Capabilities. https://pearldiverinc.com/researchinfo.html.

[19] Alluri R.K., Leland H., Heckmann N. (2016). Surgical research using national databases. Ann. Transl. Med..

[20] Schaeffer E.M., Srinivas S., Adra N., An Y., Barocas D., Bitting R., Bryce A., Chapin B., Cheng H.H., D’Amico A.V. (2022). Prostate Cancer, Version 1.2023 Featured Updates to the NCCN Guidelines. Natl. Compr. Cancer Netw..

[21] Chung J.-S., Morgan T.M., Hong S.K. (2020). Clinical implications of genomic evaluations for prostate cancer risk stratification, screening, and treatment: A narrative review. Prostate Int..

[22] Peabody J.W., DeMaria L.M., Tamondong-Lachica D., Florentino J., Acelajado M.C., Ouenes O., Richie J.P., Burgon T. (2017). Impact of a protein-based assay that predicts prostate cancer aggressiveness on urologists’ recommendations for active treatment or active surveillance: A randomized clinical utility trial. BMC Urol..

[23] Kornberg Z., Cooperberg M.R., Cowan J.E., Chan J.M., Shinohara K., Simko J.P., Tenggara I., Carroll P.R. (2019). A 17-Gene Genomic Prostate Score as a Predictor of Adverse Pathology in Men on Active Surveillance. J. Urol..

[24] Lin D.W., Zheng Y., McKenney J.K., Brown M.D., Lu R., Crager M., Boyer H., Tretiakova M., Brooks J.D., Dash A. (2020). 17-Gene Genomic Prostate Score Test Results in the Canary Prostate Active Surveillance Study (PASS) Cohort. J. Clin. Oncol..

[25] Eggener S., Karsh L.I., Richardson T., Shindel A.W., Lu R., Rosenberg S., Goldfischer E., Korman H., Bennett J., Newmark J. (2019). A 17-gene Panel for Prediction of Adverse Prostate Cancer Pathologic Features: Prospective Clinical Validation and Utility. Urology.

[26] Murphy A.B., Abern M.R., Liu L., Wang H., Hollowell C.M.P., Sharifi R., Vidal P., Kajdacsy-Balla A., Sekosan M., Ferrer K. (2021). Impact of a Genomic Test on Treatment Decision in a Predominantly African American Population With Favorable-Risk Prostate Cancer: A Randomized Trial. J. Clin. Oncol..

[27] Eymech O., Brunckhorst O., Fox L., Jawaid A., Van Hemelrijck M., Stewart R., Dasgupta P., Ahmed K. (2022). An exploration of wellbeing in men diagnosed with prostate cancer undergoing active surveillance: A qualitative study. Support. Care Cancer.

[28] Eure G., Germany R., Given R., Lu R., Shindel A.W., Rothney M., Glowacki R., Henderson J., Richardson T., Goldfischer E. (2017). Use of a 17-Gene Prognostic Assay in Contemporary Urologic Practice: Results of an Interim Analysis in an Observational Cohort. Urology.

[29] Badani K.K., Kemeter M.J., Febbo P.G., Lawrence H.J., Denes B.S., Rothney M.P., Rothberg M.B., Brown G.A. (2015). The Impact of a Biopsy Based 17-Gene Genomic Prostate Score on Treatment Recommendations in Men with Newly Diagnosed Clinically Prostate Cancer Who are Candidates for Active Surveillance. Urol. Pract..

[30] Herlemann A., Huang H.-C., Alam R., Tosoian J.J., Kim H.L., Klein E.A., Simko J.P., Chan J.M., Lane B.R., Davis J.W. (2019). Decipher identifies men with otherwise clinically favorable-intermediate risk disease who may not be good candidates for active surveillance. Prostate Cancer Prostatic Dis..

[31] Tosoian J.J., Chappidi M.R., Bishoff J.T., Freedland S.J., Reid J., Brawer M., Stone S., Schlomm T., Ross A.E. (2017). Prognostic utility of biopsy-derived cell cycle progression score in patients with National Comprehensive Cancer Network low-risk prostate cancer undergoing radical prostatectomy: Implications for treatment guidance. BJU Int..

[32] Jo J.K., Hong S.K., Byun S.S., Zargar H., Autorino R., Lee S.E. (2017). Positive surgical margin in robot-assisted radical prostatec-tomy: Correlation with pathology findings and risk of biochemical recurrence. Minerva Urol. Nephrol..

[33] Parker C.C., Clarke N.W., Cook A.D., Kynaston H.G., Petersen P.M., Catton C., Cross W., Logue J., Parulekar W., Payne H. (2020). Timing of radiotherapy after radical prostatectomy (RADICALS-RT): A randomised, controlled phase 3 trial. Lancet.

[34] Morgan T.M., Hawken S.R., Ghani K.R., Miller D.C., Feng F.Y., Linsell S.M., A Salisz J., Gao Y., E Montie J., Cher M.L. (2016). Variation in the use of postoperative radiotherapy among high-risk patients following radical prostatectomy. Prostate Cancer Prostatic Dis..

[35] Suardi N., Gallina A., Lista G., Gandaglia G., Abdollah F., Capitanio U., Dell’oglio P., Nini A., Salonia A., Montorsi F. (2013). Impact of Adjuvant Radiation Therapy on Urinary Continence Recovery After Radical Prostatectomy. Eur. Urol..

[36] Pra A.D., Ghadjar P., Hayoz S., Liu V., Spratt D., Thompson D., Davicioni E., Huang H.-C., Zhao X., Liu Y. (2022). Validation of the Decipher genomic classifier in patients receiving salvage radiotherapy without hormone therapy after radical prostatectomy—An ancillary study of the SAKK 09/10 randomized clinical trial. Ann. Oncol..

[37] Spratt D.E., Yousefi K., Deheshi S., Ross A.E., Den R.B., Schaeffer E.M., Trock B.J., Zhang J., Glass A.G., Dicker A.P. (2017). Individual Patient-Level Meta-Analysis of the Performance of the Decipher Genomic Classifier in High-Risk Men After Prostatectomy to Predict Development of Metastatic Disease. J. Clin. Oncol..

[38] Dalela D., Santiago-Jiménez M., Yousefi K., Karnes R.J., Ross A.E., Den R.B., Freedland S.J., Schaeffer E.M., Dicker A.P., Menon M. (2017). Genomic Classifier Augments the Role of Pathological Features in Identifying Optimal Candidates for Adjuvant Radiation Therapy in Patients With Prostate Cancer: Development and Internal Validation of a Multivariable Prognostic Model. J. Clin. Oncol..

[39] NRG Oncology Parallel Phase III Randomized Trials of Genomic-Risk Stratified Unfavorable Intermediate Risk Prostate Cancer: De-Intensification and Intensification Clinical Trial Evaluation (Guidance). https://www.nrgoncology.org/Clinical-Trials/Protocol/nrg-gu010-1?filter=nrg-gu010-1.

[40] NRG Oncology Parallel Phase III Randomized Trials for High Risk Prostate Cancer Evaluating De-Intensification for Lower Genomic Risk and Intensification of Concurrent Therapy for Higher Genomic Risk with Radiation (Predict-RT*). https://www.nrgoncology.org/Clinical-Trials/Protocol/nrg-gu009-1?filter=nrg-gu009-1.

[41] Cuzick J., Berney D.M., Fisher G., Mesher D., Møller H., Reid J.E., Perry M., Park J., Younus A., Gutin A. (2012). Prognostic value of a cell cycle progression signature for prostate cancer death in a conservatively managed needle biopsy cohort. Br. J. Cancer.

[42] Bishoff J.T., Freedland S.J., Gerber L., Tennstedt P., Reid J., Welbourn W., Graefen M., Sangale Z., Tikishvili E., Park J. (2014). Prognostic Utility of the Cell Cycle Progression Score Generated from Biopsy in Men Treated with Prostatectomy. J. Urol..

[43] Saad F., Latour M., Lattouf J.-B., Widmer H., Zorn K.C., Mes-Masson A.-M., Ouellet V., Saad G., Prakash A., Choudhury S. (2017). Biopsy Based Proteomic Assay Predicts Risk of Biochemical Recurrence after Radical Prostatectomy. J. Urol..

